# Non-operative treatment strategy for appendiceal abscess in children under 3 years old: a retrospective observational study

**DOI:** 10.3389/fped.2023.1234820

**Published:** 2023-10-25

**Authors:** Huan Li, Jianfeng Luo, Haibin Wang, Qin Guo, Peng Huang, Haiyan Lei, Wenhai Li, Jun Yang

**Affiliations:** Department of General Surgery, Wuhan Children's Hospital (Wuhan Maternal and Child Healthcare Hospital), Tongji Medical College, Huazhong University of Science & Technology, Wuhan, China

**Keywords:** appendiceal abscess, non-surgical treatment, children, appendicolith, appendiceal phlegmon

## Abstract

**Background:**

There are few studies on appendiceal abscess with appendicolith in children under 3 years old. This study aims to explore the success rate of non-surgical treatment of appendiceal abscess and assess the potential influence of an appendicolith on non-surgical treatment outcomes in children under 3 years old.

**Methods:**

The clinical data of children under 3 years old who were diagnosed with appendiceal abscess at the Wuhan Children's Hospital, China, from February 2013 to May 2020 were collected. According to the findings of ultrasonography and CT imaging, they were divided into two groups, namely, the appendicolith group and the non-appendicolith group.

**Results:**

A total of 94 children with appendiceal abscess were identified, meeting the specified study criteria, and categorized into two groups, namely, the appendicolith group (*n* = 51, 54.3%) and the non-appendicolith group (*n* = 43, 45.7%). Non-surgical treatment was unsuccessful in six out of the 94 children, yielding an overall success rate of 93.6% for non-surgical management of appendiceal abscess in children under 3 years old. The success rate for non-surgical treatment in the appendicolith subgroup was 90.2%, whereas that for the non-appendicolith subgroup was 97.7%. No statistically significant distinction was observed between the two groups (*P *= 0.292). Likewise, there were no significant differences in gender, age, duration of symptoms, fever, vomiting, diarrhea, rebound pain, white blood cell count, C-reactive protein level, and abscess cross-sectional area between the appendicolith group and the non-appendicolith group. However, there is a statistical difference in tenderness in the right lower abdomen.

**Conclusion:**

Non-surgical treatment of appendiceal abscess has a high success rate and can be considered an effective treatment strategy. In pediatric patients under 3 years old without evidence of complete intestinal obstruction or diffuse peritonitis, non-surgical treatment may be considered for appendiceal abscess.

## Introduction

Acute appendicitis is a common pediatric surgical emergency, accounting for about 1%–8% of all abdominal pain cases (1–4). The misdiagnosis rate tends to be higher in younger children, and the risk of encountering complicated appendicitis increases by approximately fivefold (5). The manifestation of atypical appendicitis symptoms in children under 3 years old, coupled with their limited capacity for accurate communication, results in a scenario where 33%–50% of pediatric cases of acute appendicitis progress to appendiceal abscess upon admission (6, 7). Currently, the conservative management approach to appendiceal abscess entails an appendectomy performed 10–12 weeks after the initial presentation. Earlier studies have indicated a potential link between the presence of an appendicolith and heightened rates of conservative treatment failure for appendiceal abscess, prompting some scholars to propose consideration of more prompt surgical intervention (8). However, contrasting investigations have reported no discernible influence of appendicolith presence on the effectiveness of conservative treatment for appendiceal abscess (9). Nevertheless, these studies primarily centered on adults and older children, with limited attention directed toward appendiceal abscess cases featuring appendicolith in children under 3 years old. Therefore, this study aims to explore the efficacy of conservative treatment for an appendiceal abscess in children under 3 years old and to ascertain whether the presence of an appendicolith impacts the success of conservative treatment within this specific pediatric subgroup.

## Methods

All methods were conducted in accordance with the applicable guidelines and regulations, and the study protocol was reviewed and received approval from the Ethics Committee of the Wuhan Children's Hospital, China. Upon diagnosing a child with appendiceal abscess during the initial admission, comprehensive information regarding the study, associated risks, and potential benefits was communicated to the parents. Written informed consent was then obtained voluntarily for both participation and data utilization. The parents were assured that declining participation would not impact the medical care provided to their children. The study conforms to the Strengthening the Reporting of Observational Studies in Epidemiology (STROBE) guidelines (10).

A retrospective analysis was carried out on clinical data of 101 children under 3 years old, who had been diagnosed with appendiceal abscess at the Wuhan Children's Hospital affiliated with the Tongji Medical College of Huazhong University of Science and Technology from February 2013 to May 2020. There was no formal sample size calculation for this study, and we included patients retrospectively until we reached an arbitrary sample size of 100 children. This analysis encompassed demographic characteristics, treatment method, length of stay (LOS), duration of symptoms, and the presence of symptoms such as diarrhea, fever, and vomiting. In addition, signs including tenderness and rebound pain, along with measurements such as white blood cell (WBC) count and C-reactive protein (CRP) level, were reviewed. The radiological results from computed tomography (CT) or ultrasonography (US) examination were also considered. The areas of appendiceal inflammation were estimated by multiplying the maximal transverse and longitudinal dimensions as reported on hospital US or CT imaging records.

In an appendiceal abscess, a CT scan can show irregular low-density mass around the appendix, partial air accumulation, and blurred fat space (11). The US can show an irregular mixed echo mass in the ileocecal region, and a dark liquid area with poor sound transmission can be observed inside the mass. Appendicolith was defined as the presence of a high-density shadow or hyperechoic area in an appendiceal cavity or appendiceal abscess cavity by CT or US.

The cases characterized by poor general conditions, diffuse peritonitis, and complete intestinal obstruction necessitating immediate surgical intervention were excluded from the study. Conversely, other cases were directed toward non-surgical treatment. The cases in which appendectomy was performed during non-surgical treatment or cases requiring rehospitalization due to post-discharge abdominal pain within 1 month were classified as non-surgical treatment failures. The non-surgical treatment approach predominantly comprised intravenous administration of broad-spectrum antibiotics, such as metronidazole combined with ceftazidime. For the cases featuring a large abscess cavity surrounding the appendix (generally larger than 3 cm × 3 cm), percutaneous ultrasound-guided drainage was considered. During non-surgical treatment, a fluid diet was given priority, and the US was repeated every 3–4 days. In instances where non-surgical treatment yielded unsatisfactory results—manifesting as persisting abdominal pain, the onset of diffuse peritonitis, or the occurrence of intestinal obstruction—surgical intervention would be pursued. Children demonstrating an absence of symptoms such as fever, abdominal pain, tenderness, and rebound pain, alongside a notable reduction in the size of the appendix abscess cavity, coupled with the normalization of WBC and CRP, would be considered candidates for discharge. But if the diameter of the appendiceal abscess remained greater than 2 cm at the point of discharge, we would recommend continued oral third-generation cephalosporins such as cefixime for at least 1-week post-discharge.

### Statistical analysis

A total of seven pediatric patients necessitating emergency surgery after admission were excluded from the study. Descriptive statistics for quantitative variables such as age, hospital LOS, symptom duration, WBC count, CRP level, and the area of inflammation were presented in the format of mean ± standard deviation (SD). Meanwhile, categorical data, such as gender; symptoms such as diarrhea, fever, and vomiting; signs such as tenderness and rebound pain; and cases involving percutaneous ultrasound-guided drainage, were conveyed in terms of case count and percentage. The enrolled cases were categorized into two groups, namely, the appendicolith group and the non-appendicolith group. Statistical analysis was performed on the aforementioned parameters. A statistical analysis encompassed the utilization of independent sample *t*-tests for quantitative data and chi-square tests for the analysis of classified data. For the analytical process, the SPSS 26.0 statistical software was employed. In instances where the calculated *P*-value is <0.05, the presence of a statistically significant difference was established.

## Results

**Figure 1:**
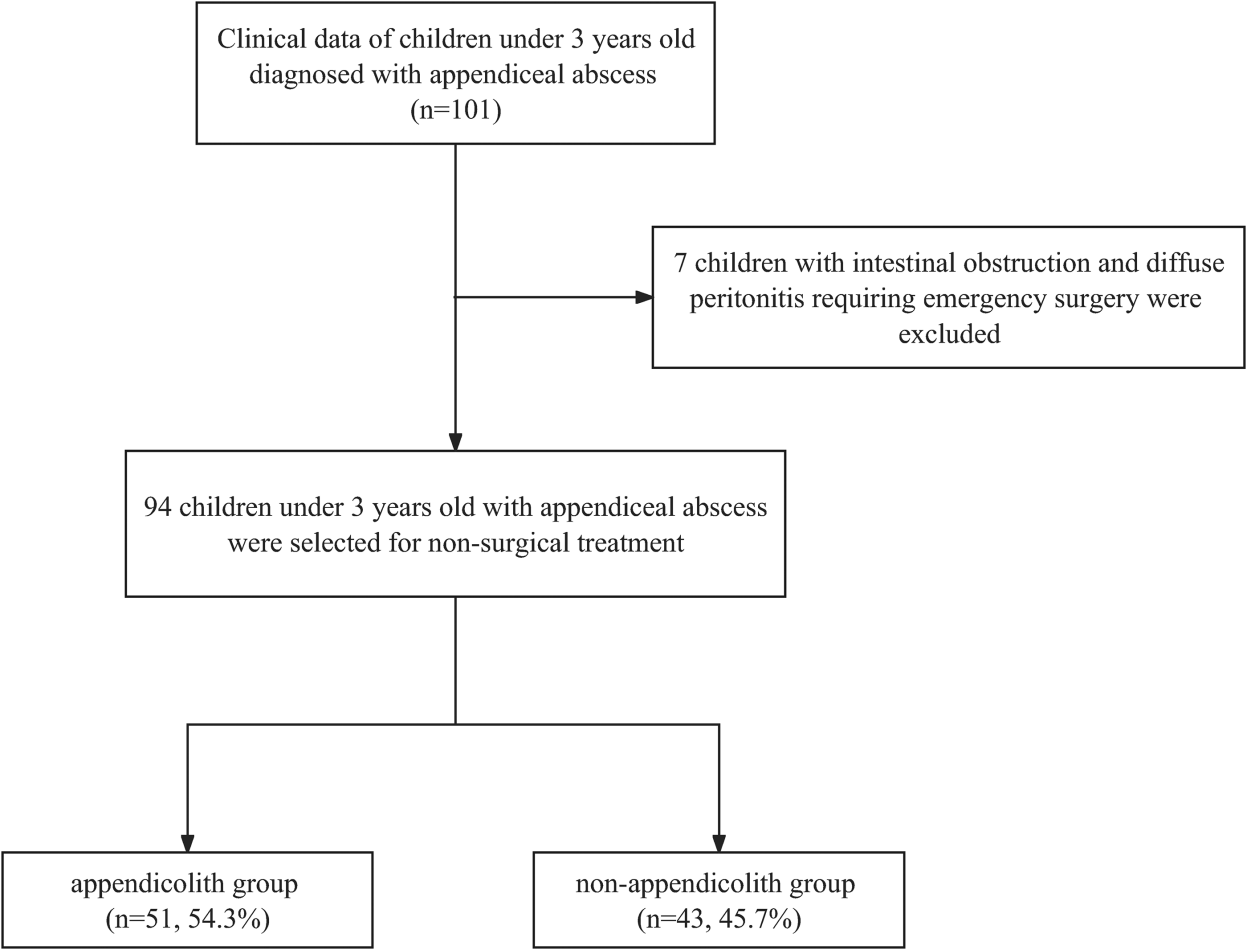
A flowchart of inclusions and exclusions.

The clinical data of 101 children under 3 years old who were diagnosed with appendiceal abscess were collected. Seven children necessitating emergency surgery due to intestinal obstruction and diffuse peritonitis were systematically excluded. A total of 94 children under 3 years old with appendiceal abscess were selected for non-surgical treatment and included in the study. Regrettably, the application of non-surgical treatment proved ineffective in six children. Specifically, two children, upon admission, were diagnosed with incomplete intestinal obstruction via CT imaging. Subsequently, surgical intervention was deemed necessary after 3 and 5 days of conservative treatment, due to the aggravation of intestinal obstruction. An additional two children underwent conservative treatment; however, persistent manifestations of intermittent high fever, unrelieved abdominal pain, and deteriorating mental state prompted a decision for surgical intervention. Equally, two children were readmitted for conservative treatment within 1-month post-discharge due to abdominal pain. A schematic representation detailing the inclusions and exclusions is presented in Figure 1.

The study cohort consisted of 57 male (60.6%) and 37 female (39.4%) children, with an average age of 2.40 ± 0.49 years (0.14–3 years). The average duration of symptoms was 6.55 ± 3.62 days. Moreover, 89 children (94.6%) had a fever, whereas vomiting and diarrhea were observed in 45 children (47.8%) and 47 children (50.0%), respectively. Among the participants, 87 children(92.5%) had right lower abdominal tenderness, with 36 children (38.2%) experiencing rebound pain. WBC counts were measured at an average of 19.76 ± 6.30 × 10^9^/L, whereas CRP levels exhibited an average of 91.96 ± 59.12 mg/L. The mean inflammatory areas associated with the appendiceal abscess were measured at 17.26 ± 13.17 cm^2^. The hospital LOS exhibited an average of 13.34 ± 5.24 days. The non-surgical treatment failed in six children, and the success rate of non-surgical treatment was 93.6%.

Out of the 94 children, abdominal US findings indicated the presence of an appendiceal abscess in 51 children (54.2%), among which 19 children (37.3%) exhibited appendicolith. Meanwhile, abdominal CT scans revealed appendiceal abscess in 43 children (45.7%), with 32 of them (74.4%) displaying appendicolith. According to the US and CT findings, the pediatric patients were divided into two groups, namely, the appendicolith group (*n* = 51, 54.3%) and the non-appendicolith group (*n* = 43, 45.7%). The analysis of the data from Table 1 divulges no significant disparities in terms of gender, age, duration of symptoms, presence of fever, vomiting, diarrhea, rebound pain, WBC counts, CRP levels, or the mean inflammatory area of abscess between the aforementioned groups. The incidence of right lower abdominal tenderness in the appendicolith group (100.0%) was higher than that in the non-appendicolith group (83.7%), and there was a statistical difference between the two groups (*P* = 0.009). Notably, the success rate for non-surgical treatment in the appendicolith group stood at 90.2%, while the non-appendicolith group exhibited a success rate of 97.7%. There was no significant statistical difference between the two groups (*P *= 0.292).

**Table 1:** Comparison of clinical data of children with and without appendicolith.

	Appendicolith (*n* = 51)	No appendicolith (*n* = 43)	*P*-value
Sex			
Male	31 (60.8%)	26 (60.5%)	0.975
Female	20 (39.2%)	17 (39.5%)	
Age (years)	2.39 ± 0.45	2.41 ± 0.54	0.822
LOS (days)	13.35 ± 5.52	13.33 ± 4.96	0.980
Duration of symptoms (days)	6.02 ± 2.37	7.26 ± 4.61	0.098
Fever	49 (96.1%)	40 (93.0%)	0.844
Vomiting	24 (47.1%)	21 (48.8%)	0.863
Diarrhea	27 (52.9%)	20 (46.5%)	0.535
Tenderness	51 (100%)	36 (83.7%)	0.009^a^
Localized peritonitis	21 (41.2%)	15 (34.9%)	0.532
WBC	20.59 ± 6.04	18.78 ± 6.53	0.166
CRP	98.66 ± 59.22	83.82 ± 58.66	0.227
Inflammatory area (mm^2^)	16.69 ± 15.46	14.72 ± 12.21	0.500
Percutaneous ultrasound-guided drainage	11 (21.6%)	9 (20.9%)	0.940
Overall success	46 (90.2%)	42 (97.7%)	0.292

The overall success rates of the two groups were 90.2% and 97.7%, respectively, with no statistically significant difference.

^a^ Only tenderness was found to be statistically different between the two groups (*P* < 0.05).

A total of 101 clinical datasets of pediatric patients under 3 years old with appendiceal abscess were gathered. Among them, seven pediatric patients, who did not meet the inclusion criteria, were subsequently excluded. This left 51 pediatric patients with appendicoliths and 43 pediatric patients without appendicoliths in the clinical datasets of pediatric patients with appendiceal abscess.

## Discussion

Acute appendicitis holds a substantial incidence rate of 7%–9% within the human population (12), with its peak occurrence primarily observed between the ages of 10 and 19. However, the prevalence of appendicitis among children under 3 years old is notably lower, ranging from 2.3% to 5.4% (13, 14). Due to the challenges posed by the limited ability of young children to articulate their symptoms accurately, coupled with the presence of atypical clinical presentations of appendicitis, medical professionals occasionally encounter diagnostic difficulties. This situation often contributes to the occurrence of appendiceal perforation at the initial diagnosis, ranging from 30% to 75% in children (15–20), with children under 3 years old displaying a particularly high appendiceal perforation rate exceeding 90% (17, 18). The low incidence but high perforation rate of appendicitis in young children under 3 years old leads to 33%–50% of pediatric patients being diagnosed with appendiceal abscess upon presentation (6, 7, 21).

In the realm of acute appendicitis in older children, the occurrence of diarrhea is a seldom reported symptom. However, among children under 3 years old afflicted with appendicitis, the prevalence of diarrhea ranges from 33% to 46% (22–24), and the clinical manifestations include vomiting, abdominal pain, and fever (13). A noteworthy correlation exists between the occurrence of influenza and appendicitis, with research revealing a certain interrelation between viral diseases and appendicitis (25). The underdeveloped anatomical structure of children, coupled with an inadequate omental presence, renders the appendix susceptible to rapid perforation. This vulnerability is accentuated by rectal mucosal irritation, leading to a heightened prominence of frequent diarrhea. Perforation of the appendix and leakage of intestinal contents often leads to irritation of the rectal mucosa, resulting in frequent diarrhea in young children with appendicitis; hence, gastroenteritis has become the most common misdiagnosis of appendicitis in this age group (15, 16, 26). In this study, the incidence of diarrhea in children was 50%, which was similar to that in the findings of other researchers.

Appendectomy, particularly laparoscopic appendectomy, stands as the prevalent and established approach for addressing acute appendicitis (27, 28). However, there is still controversy in the medical community about the optimal strategy for appendiceal abscess. Fagenholz et al. (29) categorized appendiceal abscess into three grades considering the abscess size measured by CT scan and its interaction with adjacent tissues. These are as follows: grade I for abscess diameter of <3 cm; grade II for well-encapsulated abscesses with a clear shell formation, encompassing the appendix, and abscess diameter of >3 cm; and grade III for expansive appendiceal abscesses not restricted in scope, extending to the intestinal septa, pelvic cavity, and retroperitoneal region. Children with grade III appendiceal abscess usually have poor general conditions, such as severe abdominal pain, recurrent fever, and poor intestinal function. Such cases often prove refractory to conservative treatment. To thwart the progression toward sepsis and septic shock, prompt surgical intervention becomes imperative, ideally within 24 h of the initial diagnosis and evaluation (30). Conversely, children with grade I and II appendiceal abscesses exhibit a more favorable clinical status. While some experts advocate early surgery, numerous studies demonstrate a notable escalation in intraoperative and postoperative complications (31, 32). At present, a prevailing approach advocates initial non-surgical management and subsequent appendectomy deferred until 10–12 weeks after symptom alleviation. Some studies have shown that the incidence of postoperative wound infection and intestinal obstruction in children with delayed appendectomy is lower than that in children with early emergency appendectomy, and they are hospitalized less due to abdominal pain again, which is safe and effective for most children (32). The World Society of Emergency Surgery (WESE) similarly advocates the early conservative treatment of appendiceal abscess (33). Notably, while the aforementioned studies primarily pertain to adults and older children, systematic investigation of conservative treatment for appendiceal abscess in children under 3 years old remains scant.

In this study, 101 children with appendiceal abscess were collected. Seven children who had intestinal obstruction or diffuse peritonitis at admission and received surgical treatment were excluded. All pediatric patients of the rest were scheduled for interval appendectomies regardless of appendicolith status. Although non-surgical management was pursued initially, an appendectomy was planned after the acute inflammation resolved. This approach accounts for the known risks of recurrence and complications associated with retained appendicoliths. Allowing the acute inflammation to subside prior to surgery balances the avoidance of urgent surgery and its risks against the need for ultimate appendiceal removal in those with appendicoliths.

Non-surgical treatment was unsuccessful in six children. Two children with appendicolith were admitted to the hospital due to abdominal pain within 1 month after receiving anti-infective treatment. Four children required surgical intervention due to intestinal obstruction or inadequate long-term infection control during conservative treatment. The success rate of conservative treatment stood at 93.6%, aligning closely with findings from earlier research. Success rates for conservative treatment of appendiceal abscess among both adults and children have ranged from 84% to 98% (34, 35). All of our non-surgical pediatric patients had grade I or II abscesses, which may have contributed to the high non-operative success rate. Meanwhile, the inclusion of children who underwent percutaneous ultrasound-guided drainage could potentially impact the overall success and failure rates.

Acute appendicitis is widely recognized as an inflammatory response resulting from an excessive bacterial proliferation after obstruction of the appendix lumen, often attributed to the presence of an appendicolith (36, 37). Studies have shown that appendicolith in children is more likely to cause appendix lumen obstruction than that in adults and is an important influencing factor for appendix perforation (38, 39). Some researchers posit that the presence of appendicolith will increase the failure rate of conservative treatment for appendiceal abscess, thereby advocating for emergent surgical intervention (8, 28). Conversely, other scholars contend that even in cases of appendiceal abscess with appendicolith among adults and older children, a course of conservative treatment followed by delayed appendectomy remains a viable strategy (3, 9). Nevertheless, it is pertinent to note that research focused on appendiceal abscesses accompanied by appendicolith in children under 3 years old remains relatively scarce.

To investigate the impact of appendicolith on the conservative management of appendiceal abscess in children below 3 years of age, we categorized the clinical data into two groups based on the US and CT findings: the appendicolith group and the non-appendicolith group. There were no significant statistical differences in gender, age, duration of symptoms, fever, vomiting, diarrhea, rebound pain, WBC counts, CRP levels, and mean inflammatory areas between the two groups. The prevalence of right lower abdominal tenderness was notably higher in the appendicolith group (100.0%) compared to that in the non-appendicolith group (83.7%), thereby indicating a statistical discrepancy between the groups. It is essential to recognize that infants and toddlers often exhibit limited coordination and may not effectively articulate their sensations. The manifestation of intense crying and evident discomfort is a common occurrence. Consequently, accurate diagnosis of right lower abdominal tenderness is challenging and imbued with a certain degree of subjectivity. The success rate of conservative treatment in the two groups was 90.2% and 97.7%, respectively. There was no significant statistical difference between the two groups (*P *= 0.292), indicating that appendicolith has no significant effect on the conservative treatment of appendiceal abscess in children under 3 years old. These findings align with the conclusions of prior investigations (40).

Both US and CT can be used for the diagnosis and treatment of diseases in children in our country. The precision of disease diagnosis through US is intricately linked to the skill level of medical practitioners. Consequently, we opted for abdominal CT scans for the majority of the pediatric population.

Several limitations exist within the scope of this study. Primarily, it maintains a retrospective design, introducing potential selection bias. Furthermore, the study grapples with a constrained sample size due to the low prevalence of appendicitis among children under 3 years old and the exclusive focus on a single center. Consequently, the ability of this study to detect significant treatment failure rates is limited by statistical power. In addition, the identification of an appendicolith relies on imaging techniques, which exhibit inherent sensitivity constraints and might not consistently correlate with pathological findings despite their utility. A multicenter prospective study and pathological findings should be performed in the future to improve the diagnosis and improve the limitations of this study.

## Conclusion

Non-surgical treatment of appendiceal abscess has a high success rate and can be considered an effective treatment strategy. In pediatric patients under 3 years old without evidence of complete intestinal obstruction or diffuse peritonitis, non-surgical treatment may be considered for appendiceal abscess.

## Data Availability

The raw data supporting the conclusions of this article will be made available by the authors, without undue reservation.
